# Prenatal echocardiographic diagnosis of a discontinuous left pulmonary artery with Taussig–Bing syndrome: a case report and literature review

**DOI:** 10.3389/fped.2024.1437500

**Published:** 2024-12-12

**Authors:** Yun He, Yufei Yang, Xiaoyu Song, Zhonglei Zhang, Dingfang Yan, Chao Xie, Min Zeng, Wenjun Zhang

**Affiliations:** ^1^ Department of Ultrasound Medicine, Taihe Hospital, Hubei University of Medicine, Shiyan, Hubei, China; ^2^ Department of Cardiothoracic Surgery, Taihe Hospital, Hubei University of Medicine, Shiyan, Hubei, China

**Keywords:** fetus, malformation, pulmonary artery, echocardiography, congenital heart

## Abstract

**Background:**

Unilateral pulmonary artery discontinuity (UPAD) is a rare fetal abnormality, for which a prenatal ultrasonographic diagnosis remains challenging. We report a case of left pulmonary artery discontinuity in association with Taussig–Bing syndrome, which has rarely been reported in the literature thus far.

**Case presentation:**

A pregnant woman with a fetus with congenital heart disease (CHD) at 23 weeks gestation was referred to our center. She denied any familial history of genetic disorders in either spouse, and non-invasive prenatal testing (NIPT) also showed a low risk of CHD for the fetus. An ultrasound examination revealed a complex cardiac malformation indicative of left pulmonary artery discontinuity originating from the ductus arteriosus with Taussig–Bing syndrome. The family eventually chose to terminate the pregnancy and agreed to an autopsy, which confirmed that the prenatal echocardiographic diagnosis was correct. In addition, in this report, we review and analyze 17 reported cases of prenatal echocardiographic diagnoses of UPAD.

**Conclusions:**

UPAD is characterized by discontinuity between the proximal and distal pulmonary arteries, along with a ductal origin of the distal pulmonary artery. A discontinuous pulmonary artery originating from the ductus can be detected on prenatal sonography using ultrasound technology. The aim is to detect malformations earlier and carry out the necessary intervention measures as soon as possible.

## Case presentation

**Figure 1:**
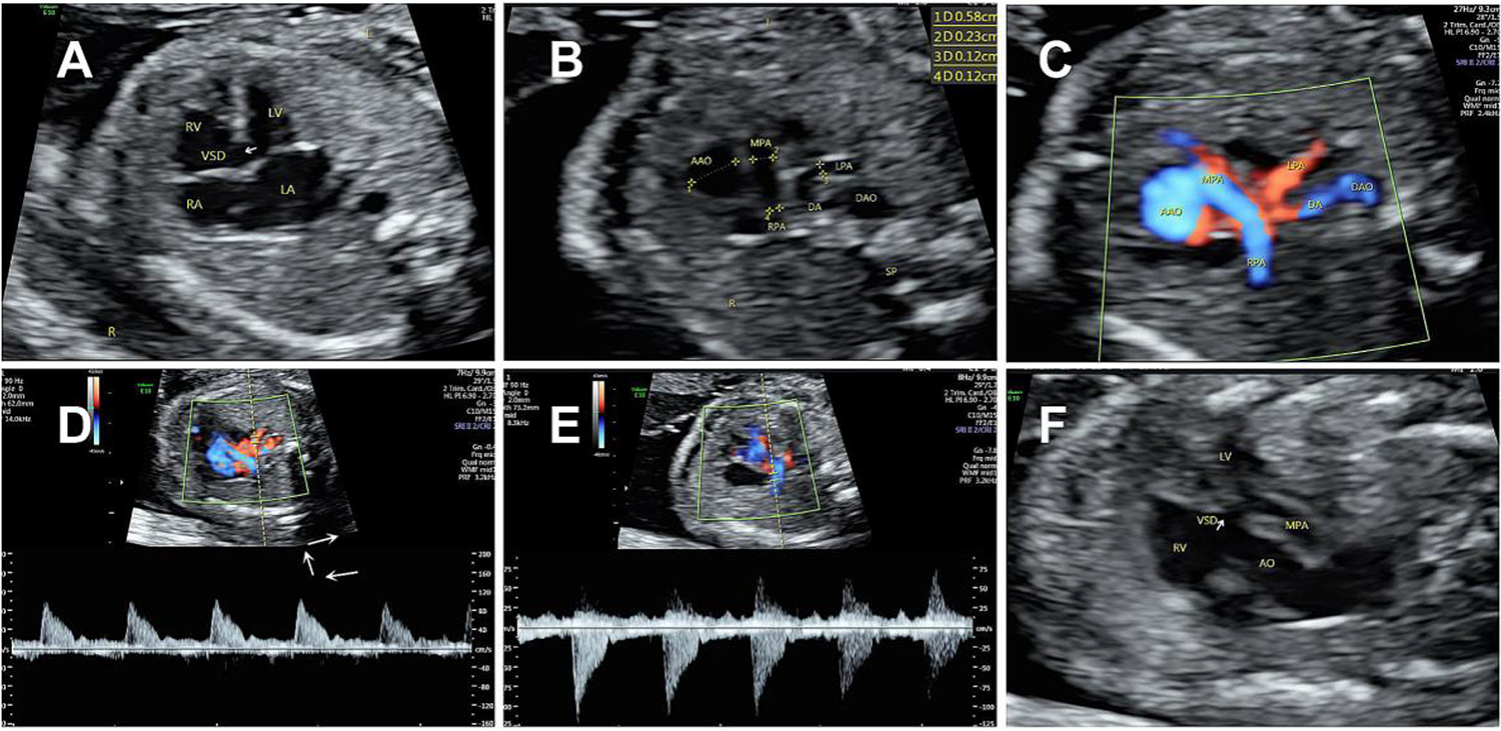
Prenatal echocardiogram showing **(A)** a large ventricular septal defect. **(B**,**C)** Main pulmonary artery (MPA-2.3 mm) only gave rise to the right pulmonary artery (RPA-1.2 mm). CDFI showed a blood flow (LPA-1.2 mm) originated from the descending aorta through the ductus arteriosus to supply the left pulmonary artery. **(D**,**E)** Spectral Doppler ultrasound showed similar high resistance waveforms of the pulmonary arteries on both sides. **(F)** A complete transposition of the aorta originating solely from the right ventricle.

A 31-year-old woman with a fetus with congenital heart disease (CHD) at 23 gestational weeks was referred to our hospital. She denied any history of non-steroidal anti-inflammatory drug (NSAID) use during the pregnancy, exposure to toxins or radiation, familial history of genetic disorders in either spouse, or congenital heart disease. Non-invasive prenatal testing (NIPT) also showed a low risk of CHD for the fetus. The fetal echocardiogram revealed an apparent ventricular septal defect (VSD) measuring 4.7 mm (Figure 1A). In the three-vessel view (3VV), compared with the aorta, there was stenosis in the pulmonary artery. To confirm this and explore why it was narrowed, we first determined the aorta by its three main branches in the long-axis view, and then, in the 3VV, slightly rotated the ultrasound beam and found that there was no bifurcation in the main pulmonary artery (MPA) and it only gave rise to the right pulmonary artery (RPA) (Figure 1B). However, color Doppler flow imaging (CDFI) showed blood flow signals originating from the descending aorta through the left side of the ductus arteriosus (DA) to supply the left pulmonary artery (LPA) (Figure 1C). Based on the CDFI results, it appeared that the LPA originated from the left side of the ductus arteriosus. Thus, vessels suspected to be the LPA were measured which corresponded to the waveform of the pulmonary artery, and the peak flow velocity in the bilateral pulmonary arteries measured 105 cm/s (left) and 107 cm/s (right) (Figures 1D,E).

When we performed a continuous scan to explore the origin of the left pulmonary artery, in the ventricular outflow tract section, we also observed the right pulmonary artery mostly originated from the right ventricle but was overriding the ventricular septum, while the aorta completely originated from the right ventricle (Figure 1F). In addition, the two major arteries were not crossed and arose from the right ventricle in parallel.

**Figure 2:**
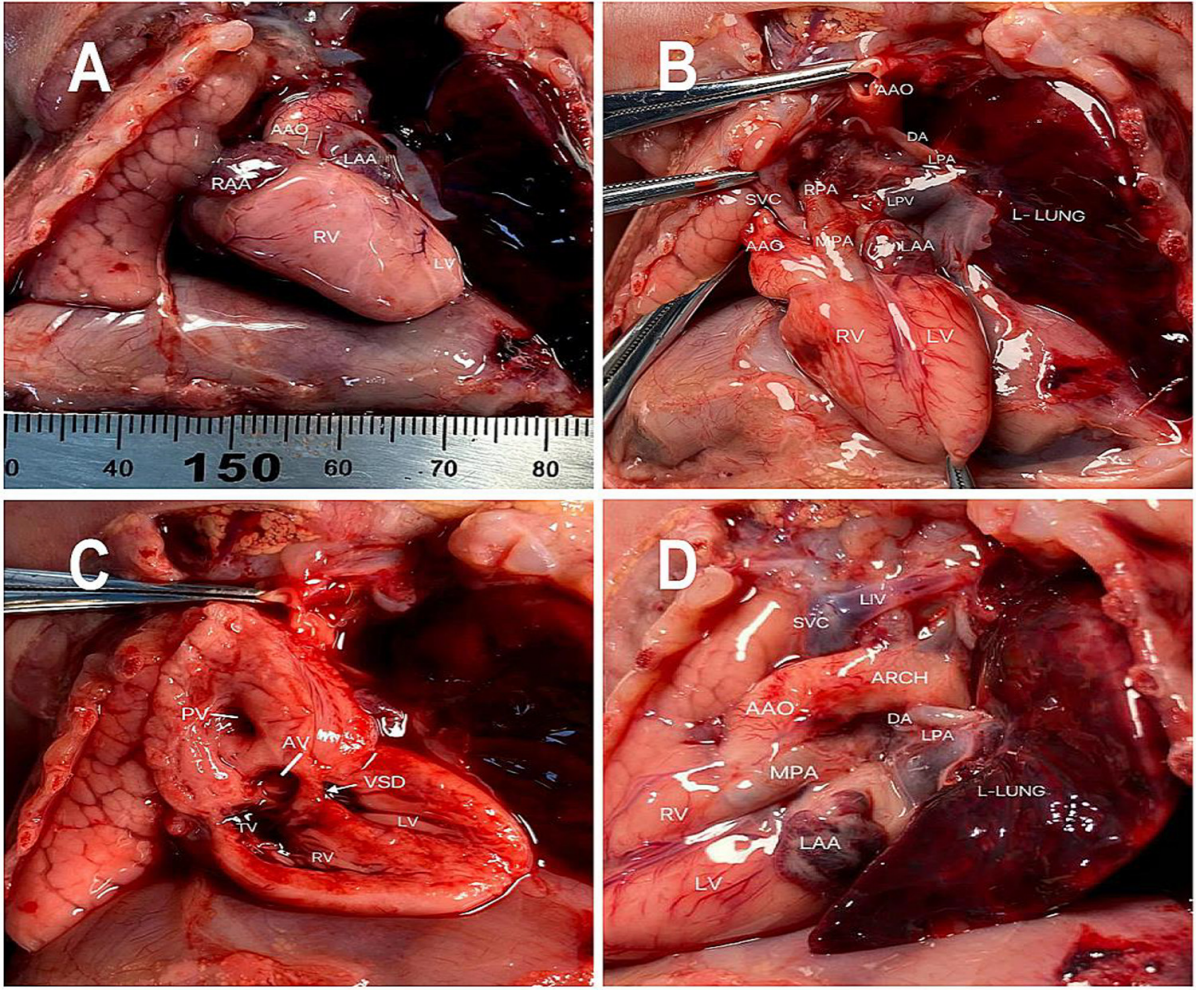
Pathological findings including **(A)** an ascending aorta arising from the right ventricle **(B)** the right pulmonary artery arising from the atretic main pulmonary artery. **(C)** Double-outlet right ventricle (DORV) with a subpulmonary ventricular septal defect **(D)** the left pulmonary artery arising from the descending aorta through the ductus arteriosus.

These findings led to a final diagnosis of Taussig–Bing syndrome associated with unilateral pulmonary artery discontinuity (UPAD) for the fetus. The Family decided to terminate the pregnancy, and the autopsy confirmed the diagnosis. The autopsy demonstrated levocardia with an ascending aorta arising from the right ventricle (Figure 2A). The main pulmonary artery arises from the right ventricle and is left and parallel to the ascending aorta (Figure 2B). The right pulmonary artery arose from the main pulmonary artery with a discontinuity of the left pulmonary artery (Figures 2B,C). The left patent ductus arteriosus supplied the left pulmonary artery (Figure 2D).

## Discussion

UPAD is a rare CHD with an estimated prevalence of 1 in 200,000 and was initially described by Fraentzel in 1868 (1, 2). It is also referred to as isolated unilateral pulmonary artery agenesis or ductal origin of distal pulmonary artery (DODPA). The former term can be misleading as an intrapulmonary vessel is present in fetuses with this condition. The term UPAD is more widely accepted and used, as it clarifies that distal and proximal pulmonary artery discontinuities can coexist. Embryologically, under normal conditions, the proximal sixth aortic arch forms the extrapulmonary pulmonary artery and joins the intrapulmonary pulmonary arteries. The majority of UPAD studies have indicated that the cause of this developmental defect is the involution of the proximal sixth aortic arch, resulting in the absence of the extrapulmonary pulmonary artery (3). Consequently, a single pulmonary artery arising from the MPA trunk can be found on ultrasound imaging, while the intrapulmonary pulmonary arteries arise normally from their respective lung buds and persistently connect with the distal sixth aortic arch. This explains why, during ultrasound scanning from the hilum, the distal segment of the pulmonary artery is normally connected to the corresponding lung tissue. Since the distal sixth aortic arch is destined to become the ductus arteriosus, the pulmonary artery always arises by way of the ductus-like collateral vessels from the aortic arch system or from the bronchial arteries. In our case, the pulmonary artery originated from the descending aortic arch through the ductus arteriosus.

Accurately determining the origination of the pulmonary artery from the aorta is crucial. If the unilateral pulmonary artery anomaly originates from the ascending aorta, it is classified as hemitruncus arteriosus or anomalous origin of one pulmonary artery from the ascending aorta (AOPA) (4). In comparison to UPAD, these anomalies typically result in life-threatening pulmonary hypertension earlier due to the pulmonary artery with anomalous origin receiving disordered and high-pressure blood perfusion from the aorta, while another pulmonary artery receives perfusion from the whole right ventricle. According to the distance between the anomalous origin of the pulmonary artery and the aortic valve, AOPA can be classified into proximal and distal types. Distinguishing the distal type from UPAD on ultrasound is challenging.

UPAD usually coexists with other CHDs but can also be present as an isolated anomaly. The occurrence of left pulmonary artery discontinuity (LPAD) in conjunction with other CHDs is more prevalent, such as tetralogy of Fallot (TOF), septal defect, persistent DA, or mirror image right aortic arch (MRAA) (5, 6). Nevertheless, rare literature documents the prenatal echocardiographic diagnosis of the left pulmonary artery originating from the ductus arteriosus coexist Taussig-Bing syndrome. In the latter syndrome, the pulmonary artery typically arises from both ventricles, while the aorta arises exclusively from the morphological right ventricle. The aorta is usually to the right, and is slightly anterior to, or side-by-side with the pulmonary artery. These two great vessels are usually parallel. They do not spiral around each other as in the normal heart. The pulmonary artery overrides the VSD (7). It should be noted that Taussig–Bing syndrome can be easily confused with transposition of the great arteries (TGAs).

In the prenatal stage, isolated unilateral pulmonary discontinuity can be difficult to diagnose. However, after birth, if the ductus arteriosus closes or there is vascular endothelial injury, symptoms such as coughing, hemoptysis, and recurrent respiratory infections may occur, which can potentially be life-threatening (8).

There is a consensus among sonographers who perform fetal malformation screening that conducting a continuous scan enables the acquisition of more comprehensive anatomical information (9). In challenging cases where identification of the origin of the ipsilateral pulmonary artery proves difficult, it is recommended to perform upstream scanning from the hilum to locate its origin and course. Furthermore, we utilized spectral Doppler ultrasound to measure the spectrum of the pulmonary arteries on both sides and observed similar waveforms. Therefore, spectral Doppler ultrasound can be a valuable tool for assisting in the diagnosis of undiagnosed (but suspected) vessels with abnormal origin. Identifying the origin of the pulmonary arteries should be considered routine prenatal screening to prevent missed detection of various anomalous origins such as AOPA, pulmonary artery sling, and crossed pulmonary arteries (CPA), among others that are often challenging to identify using conventional views. Currently, new technology provides better support for diagnosis, for example, Chen et al. (10) used four-dimensional spatiotemporal image correlation (STIC-HD) live flow technology to acquire an overall perspective of all blood vessels, which can help physicians to have a clear depiction of great vessels in a fetus (11).

Over the past decade, the frequency of intrauterine detection of UPAD has been gradually increasing. However, it is more commonly detected in children and young adults due to clinical symptoms rather than prenatal diagnosis (12, 13). We conducted a comprehensive analysis of the existing English literature on identified fetal UPAD in PubMed within the past decade. The available research encompassed a mere five studies, comprising a total of 16 documented cases. Table 1 provides the information about each case report. From the data in Table 1, it can be observed that 5 (30%) out of the 17 patients had isolated UPAD with no other cardiac malformations. In addition, in 12 (70%) cases, UPAD was associated with other congenital heart diseases, most commonly ventricular septal defect (*n* = 6), followed by mirror image right aortic arch (*n* = 4), double-outlet right ventricle (*n* = 2), tetralogy of Fallot (*n* = 1), Taussig–Bing syndrome (*n* = 1), and persistent left superior vena cava (PLSVC) (*n* = 1). In this group, isolated right pulmonary artery discontinuity (RPAD) was less common than LPAD with CHD, accounting for 30% of all cases. Among the 10 LPAD cases, 6 (60%) were not associated with MRAA, which contradicts the finding of Li et al. (15) that the proximal pulmonary artery was found to be absent on the contralateral side of the aortic arch. However, it is worth noting that MRAA has never been found in cases of RPAD. Isolated RPAD accounted for approximately 70% of all the RPAD cases, and only two cases of RPAD were associated with other CHDs, namely, a small ventricular defect and PLSVC.

**Table 1:** Prenatal ultrasonographic manifestations and follow-up results of 17 cases of unilateral pulmonary artery discontinuity.

Years	Author	Case	Gestational age (weeks)	Affected PA	Associated heart anomalies	Outcome
2022	Chen (10)	1	25^+3^	RPA	None	Full-term delivery, surgery, alive
		2	23	LPA	MRAA	TOP, confirm end by autopsy
		3	24	RPA	VSD	Full-term delivery, surgery, alive
2021	Cha (14)	4	26	LPA	PA, VSD	Full-term delivery, surgery (RVOT reconstruction and pulmonary artery reimplantation), died (right heart failure)
2020	Wenxiu (15)	5	27	RPA	None	Full-term delivery, PDO, alive
		6	28	RPA	None	TOP, confirmed by autopsy
		7	25	RPA	None	Full-term delivery, without surgery
		8	25	RPA	None	Full-term delivery, without surgery
		9	25	LPA	MRAA	Full-term delivery, without surgery
		10	26	LPA	TOF, MRAA	Full-term delivery, TOF surgery, alive
2018	Han (12)	11	22 ^+ 2^	RPA	PLSVC	TOP, confirmed by vascular casting
	Li (16)	12	24	LPA	RIM, SV pulmonary artery, IVC and LHV connected to the left atrium, MHV and DV and RHV connected to the right atrium	Prenatal magnetic resonance imaging, TOP, confirmed by autopsy
		13	27^+1^	LPA	PS, MRAA, sinistrocardia	TOP, confirmed by autopsy
		14	24^+3^	LPA	SA, VSD, DORV, PS	TOP, confirmed by autopsy
		15	22^+2^	LPA	VSD, DORV, PS	TOP, confirmed by autopsy
		16	23^+2^	LPA	PA, VSD	TOP, confirmed by autopsy
Current	He	17	23	LPA	Taussig–Bing syndrome	TOP, confirmed by autopsy

Only first author of each study is given.

TOP, termination of pregnancy; VSD, ventricular septal defect; PA, pulmonary atresia; DORV, double-outlet right ventricle; PDO, patent ductus arteriosus occlusion; PLSVC, persistent left superior vena cava; MRAA, mirror image right aortic arch; RVOT, right ventricular outflow tract; SV, single ventricle; PS, pulmonary artery stenosis; SA, single atrium; atrioventricular septal defect; RIM, right isomerism; IVC, inferior vena cava; LHV, left hepatic vein; MHV, middle hepatic vein; RHV, right hepatic vein; DV, ductus venosus.

UPAD refers to the involution of the sixth aortic arch resulting in discontinuity between the proximal and distal pulmonary arteries, along with a ductal origin of the distal pulmonary artery. LPAD often coexists with complex congenital heart defects, while RPAD can be easily overlooked during routine scans. Therefore, whether managing complex CHD or conducting regular antenatal examinations, assessing the branching relationship of the pulmonary arteries in a three-vessel view becomes essential for identifying any abnormalities in their origins. Ultrasound is the preferred method for antenatal examination, allowing for more accurate and early detection of lesions. However, achieving this requires sonographers to possess a deeper understanding of basic views and a broader comprehension of diseases. This case emphasizes the importance of examining the continuity of the pulmonary arteries when detecting CHD as it significantly aids later-stage treatment. Furthermore, it is a reminder that LPAD can coexist with Taussig–Bing syndrome equally.

## Data Availability

The original contributions presented in the study are included in the article/Supplementary Material, further inquiries can be directed to the corresponding author.
