# Erratum: Prenatal echocardiographic diagnosis of a discontinuous left pulmonary artery with Taussig–Bing syndrome: a case report and literature review

**DOI:** 10.3389/fped.2025.1620408

**Published:** 2025-05-14

**Authors:** Frontiers Production Office

**Affiliations:** Frontiers Media SA, Lausanne, Switzerland

**Keywords:** fetus, malformation, pulmonary artery, echocardiography, congenital heart

An Erratum on Prenatal echocardiographic diagnosis of a discontinuous left pulmonary artery with Taussig–Bing syndrome: a case report and literature review By He Y, Yang Y, Song X, Zhang Z, Yan D, Xie C, Zeng M and Zhang W (2024). Front Pediatr. 12:1437500. doi: 10.3389/fped.2024.1437500

Due to an error, reviewer Jiuann Huey Ivy Lin was incorrectly listed as the editor of this paper. The correct editor is Andrew S. Day, who retains responsibility for the editorial oversight of this manuscript.

Additionally, Andrew S. Day's affiliation is listed as University of Otago, Christchurch, Christchurch, New Zealand.

Jiuann Huey Ivy Lin remains listed as the reviewer.

The original version of this article has been updated to reflect this change.

